# Synthesis and Characterization of ZnO(MgO)-CaO-SiO_2_-P_2_O_5_ Bioglass Obtained by Sol-Gel Method in Presence of Surfactant Agent

**DOI:** 10.3390/gels7040187

**Published:** 2021-10-29

**Authors:** Cristina-Daniela Ghiţulică, Andrada-Ioana Damian-Buda, Andreia Cucuruz, Georgeta Voicu

**Affiliations:** 1 Department of Science and Engineering of Oxide Materials and Nanomaterials, Politehnica University of Bucharest, 1–7 Gh. Polizu Street, 011061 Bucharest, Romania; cristina.ghitulica@upb.ro; 2 Department of Biomaterials and Medical Devices, Politehnica University of Bucharest, 1–7 Gh. Polizu Street, 011061 Bucharest, Romania; damian.andrada99@yahoo.com

**Keywords:** bioglass, sol-gel, pore size distribution, antibacterial activity, bioactivity

## Abstract

Bioglass (BG) is a class of biomaterials increasingly approached in biomedical applications, such as in regeneration of hard tissues, due to the properties of bioactivity, osteoinductivity, osteoconductivity, but also the high rate of biodegradation, both in vitro and in vivo. The present paper addresses the obtaining of bioglasses from the ZnO(MgO)-CaO-SiO_2_-P_2_O_5_ system by the sol-gel method and the use of a surfactant to ensure a specific surface or high open porosity, starting from S53P4 bioglass (53% SiO_2_, 23% Na_2_O, 20% CaO, 4% P_2_O_5_), also known as BoneAlive^®^. The precursor powders were analyzed from the phase composition point of view by complex thermal analysis and X-ray diffraction, the vitreous powders were assessed from the compositional point of view by X-ray diffraction, morpho-structural by scanning electron microscopy, specific surface area and the pore size dimension by the Brunauer–Emmett–Teller (BET) analysis, dispersion by laser granulometry, and also cell biology and surface mineralization tests were performed by immersion in SBF (simulated body fluid). The system proposed in this paper ZnO(MgO)-CaO-SiO_2_-P_2_O_5_ was successfully obtained by sol-gel method. The results showed the higher interaction between the samples and the SBF medium for samples containing magnesium (M2) and the lowest degree of mineralization after immersion in SBF was noticed for samples containing zinc (M1). The results also prove that by incorporating different ionic species in bioglass composition—Zn^2+^ and Mg^2+^, biocompatibility and antibacterial properties will be significantly enhanced.

## 1. Introduction

Biomaterials used in biomedical applications, such as in hard tissues regeneration are materials that aim to influence the processes of repair and restoration of tissues and help the body to heal and recover. In the field of hard tissue regeneration, bioinert materials are predominantly employed, but in recent years, scientific research has focused on the use of bioactive materials due to a good interaction between tissue and implant, but also for their ability to facilitate tissue regeneration and healing. One such bioactive material, studied and widely used in recent decades, is bioglass (BG). Bioglass is a class of biomaterials increasingly approached in regeneration medicine due to its properties of bioactivity, osteoinductivity, osteoconductivity, but also to the high rate of biodegradation, both in vitro and in vivo [1,2,3].

The current directions in the field of BG development for hard tissue regeneration consist of the inclusion of certain ionic species in their composition, as well as increasing the contact surface with the living environment by making porous masses (mesoporous bioglasses—MBG) [4,5,6,7,8,9,10,11,12]. Doping of glasses can be done with different cationic species, such as bivalent ions (e.g., Mg^2+^, Sr^2+^, Zn^2+^, Cu^2+^), trivalent ions (e.g., B^3+^, Ga^3+^, Ce^3+^), or tetravalent ions (e.g., Zr^4+^) into the based SiO_2_–CaO–P_2_O_5_ system, by causing certain known effects in the body—osteogenesis, antibacterial, angiogenesis, etc. [7,8,9,10,11,12,13]. However, the literature data have highlighted that the incorporation of dopants determine the decrease of the specific surface area of powders and their porosity, in case of MBG [10,11,12,14].

Regarding the synthesis of BG, now the use of 2 main methods occurs most often: the traditional process, by melting [15,16], and the sol-gel method [17,18]. The sol-gel method, classical or modified (e.g., in the presence of reticulating agents like is citric acid, ethylenediaminetetraacetate), is intensively studied due to its advantages [19], e.g., (i) obtaining particles with surfaces approximately 2 times larger than by the traditional method; (ii) increasing the SiO_2_ content up to 90% (mol) compared to 60% (mol) by the traditional method; (iii) low cost. For MBG, the porosity, represented by a network of interconnected capillaries that communicate with the exterior, is ensured by the use of a porogenic agent (for example, glucose) in the case of the traditional method or by using surfactants, by sol-gel method [20,21].

Among the most used commercial glasses, it should be mentioned the bioglass S53P4 (53% SiO_2_, 23% Na_2_O, 20% CaO, 4% P_2_O_5_) also known as BoneAlive^®^, which, in addition to bioactivity, also shows antibacterial properties.

Starting from the facts mentioned above in this study we obtained and characterized new glass materials from the ZnO(MgO)-CaO-SiO_2_-P_2_O_5_ system, by the sol-gel method in the presence of surfactant—Pluronic F127. The surfactant was used to ensure a higher specific surface area/open porosity, with reference to the S53P4 bioglass, in the composition of which Na_2_O was totally replaced with MgO and/or ZnO. This was done taking into account that:

− from a medical point of view, inside the body, Na^+^ is equimolarly replaced by K^+^, which can lead to various dysfunctions (e.g., heart rhythm disturbance, insulin synthesis) [22]; on the other hand, Zn^2+^ can lead to improved antibacterial and anti-inflammatory activity, and the presence of Mg^2+^ can improve angiogenesis and osteogenesis;
− regarding the synthesis route, given that in the composition of the glasses Na_2_O acts as fondant (reducing the melting temperature of the precursors), in this case this is no longer necessary because the sol-gel synthesis method involves low temperatures [19].

## 2. Results and Discussions

### 2.1. Characterization of Precursor Powders

Table 1 presents the oxidic compositions of synthesized precursor powders by sol-gel method in presence of Pluronic F127.

**Figure 1:**
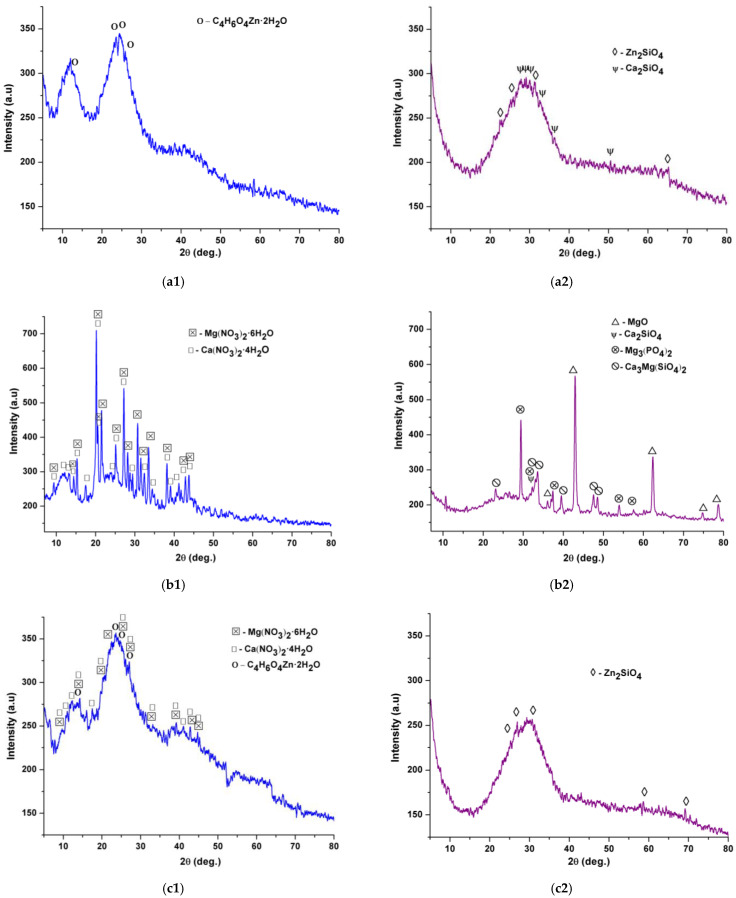
Diffractograms of dry gel precipitates ((**a1**)—M1, (**b1**)—M2, (**c1**)—M3) and calcined at 650 °C/3 h ((**a2**)—M1, (**b2**)—M2, (**c2**)—M3).

**Figure 2:**
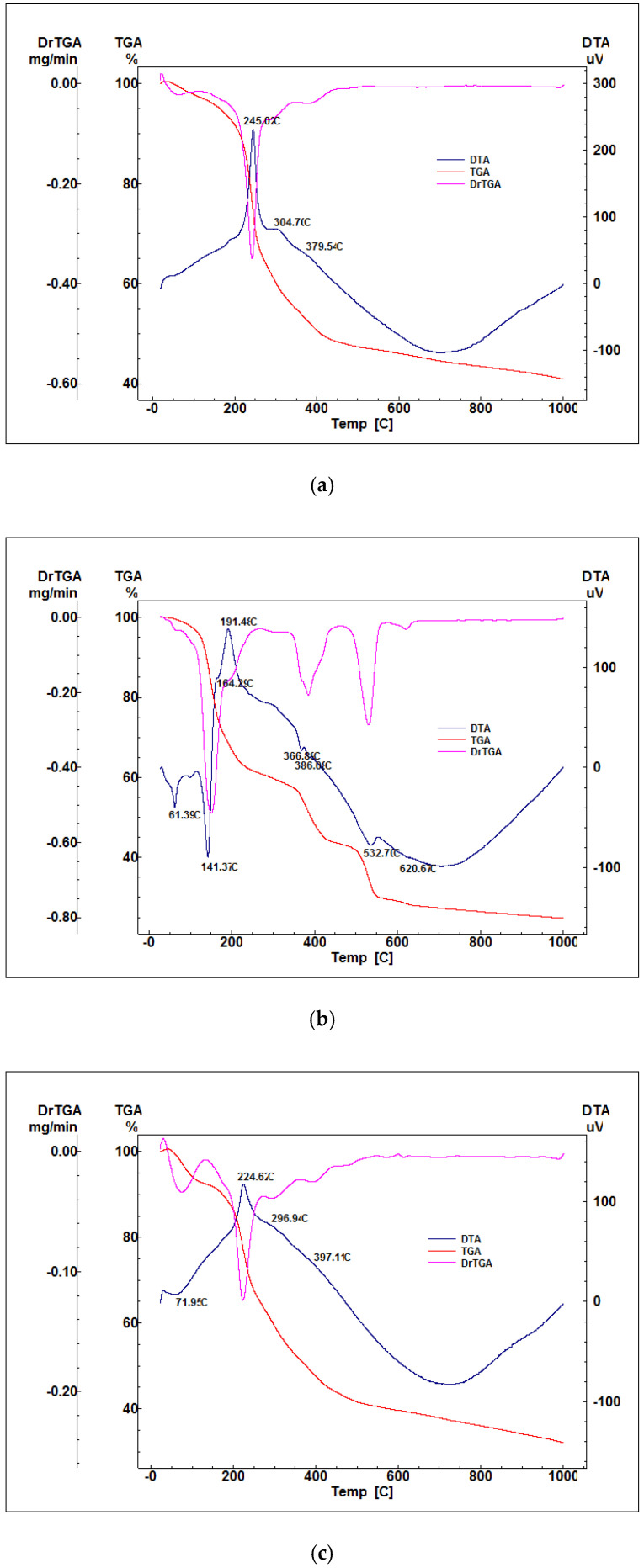
Thermal analyses of dried gel-precipitate powders: (**a**)—M1, (**b**)—M2, (**c**)—M3.

After obtaining the gel precipitates, they were dried, analyzed by X-ray diffraction, complex thermal analysis, and the obtained results are presented in Figure 1 and Figure 2.

Thus, from Figure 1a1,b1,c1, it can be seen that the dry gel precipitates show a tendency to reprecipitate the precursor salts, this process being more important in the case of masses containing Mg^2+^ (Figure 1b1,c1).

Figure 2 shows the existence of two types of thermal effects, endothermic and exothermic. From the category of endothermic effects, the decomposition of hydroxylated phases can be noticed (Zn(OH)_2_ at 110–140 °C; Mg(OH)_2_ at 360–390 °C, Ca(OH)_2_ at 450–550 °C), in the form of reprocessed nitrates or acetates (Ca(NO)_3_ nH_2_O at 500–550 °C, zinc acetate—350–400 °C) and decarbonation of carbonate phases with low crystallinity formed by the interaction of hydroxides with atmospheric CO_2_ (MgCO_3_ and CaCO_3_—650–750 °C). The strong exothermic phenomenon recorded in the range of 200–400 °C, accompanied by mass loss, is associated with the burning of the residual organic component. It is also noticed from the thermogravimetric analyzes that for all the samples synthesized in the presence of F127, there are no significant variations of the masses at temperatures above 600–650 °C. The results obtained after the characterization of the dry gel were analyzed by comparison with the data in literature to establish the optimal calcination temperature.

Therefore, the synthesized precursor powders were calcinated at 650 °C/3 h (v = 1 °C/min) in air, in order to remove the organic part, and then slowly cooled in the furnace in order to obtain the final vitreous oxide powders.

### 2.2. Characterization of Glass Powders

The powders obtained after calcination were characterized from several points of view: compositional by X-ray diffraction (XRD), morpho-structural by scanning electron microscopy (SEM), and dispersional—laser granulometry.

In Figure 1a2,b2,c2, X-ray diffractions of calcined powders are presented. Thereby, the application of heat treatment depending on the oxide composition, led to the promotion of crystallization of mineralogical phases in the form of silicates—Zn_2_SiO_4_ (JCPDS 037-1485), Ca_2_SiO_4_ (JCPDS 033-0302), Ca_3_Mg(SiO_4_)_2_ (JCPDS 035-0591), phosphate phases—Mg_3_(PO_4_)_2_ (JCPDS 048-1167), and unitary oxides—MgO (JCPDS 077-2179); in all masses, the halo in the angle range 20–40° characteristic to the vitreous masses is highlighted, but the degree of crystallinity increases with the Mg^2+^ content.

**Figure 3:**
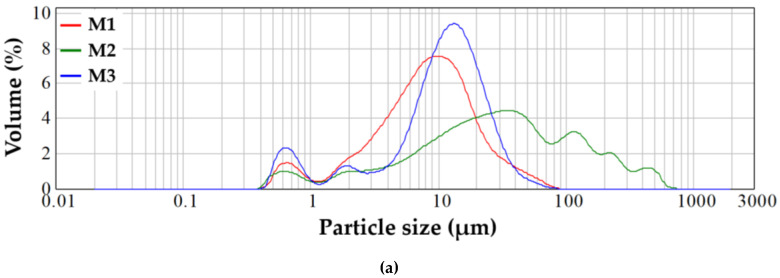

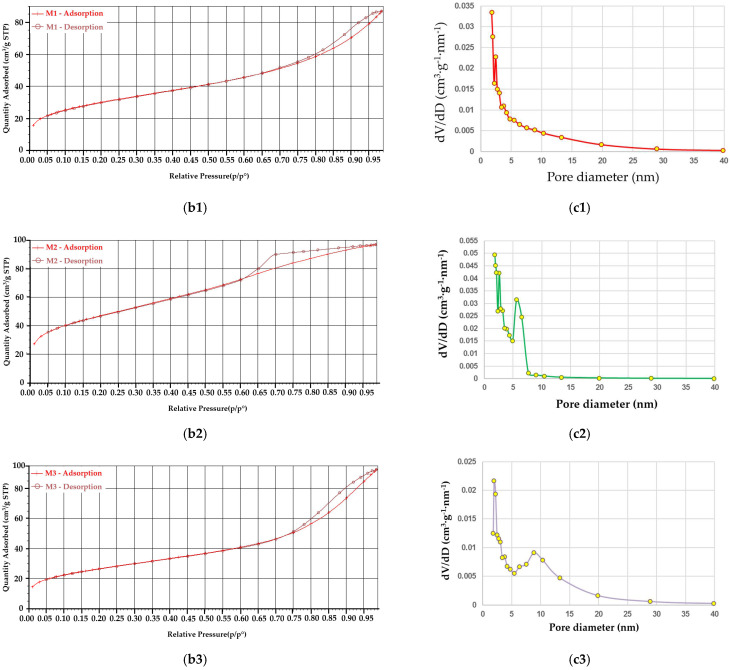
Grain size distribution (**a**), nitrogen adsorption/desorption BET isotherms ((**b1**)—M1, (**b2**)—M2, (**b3**)—M3) and pore distribution ((**c1**)—M1, (**c2**)—M2, (**c3**)—M3) for vitreous powders.

The relationship between the oxide composition of the material, the average particle size and the specific BET surface was evaluated by performing laser particle size analysis and N_2_ adsorption-desorption curves by BET method—Figure 3 and Table 2.

Thus, from Figure 3a, it can be seen that the powders show multimodal granulometric distributions, more accentuated for the masses with Mg^2+^, which can be explained by different densities of the mineralogical phases or by the agglomeration tendency of powders. Furthermore, Table 2 shows that the specific surface area of BET and the average size of the granules are larger for the masses containing Mg^2+^ compared to those containing the same proportion of ZnO, which suggests that the masses containing Mg^2+^ are characterized by mesopores. This fact is also supported by the adsorption/desorption isotherms of N_2_ (Figure 3b1,b2,b3), which are identified as a type-IV hysteresis loop and typical type—H2 and H4 hysteresis loops [23], and by the pore distribution (Figure 3c1,c2,c3) for calcined powders.

**Figure 4:**
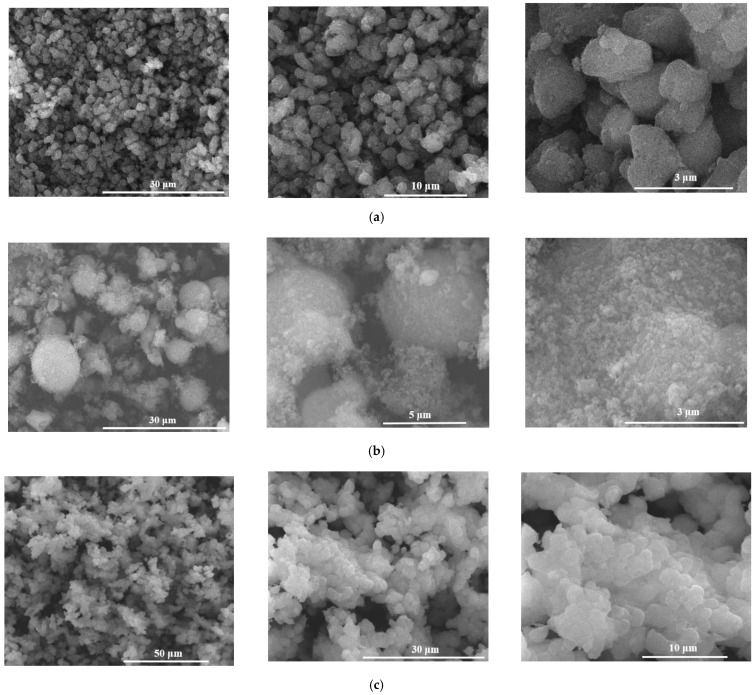
SEM images of the vitreous powders ((**a**)—M1, (**b**)—M2, (**c**)—M3).

SEM images in Figure 4 for calcined powders show that they latter are made of finer particles with approximately spherical morphology. The SEM results are in strong correlation with the laser granulometry and adsorption-desorption data of N_2_.

**Figure 5:**
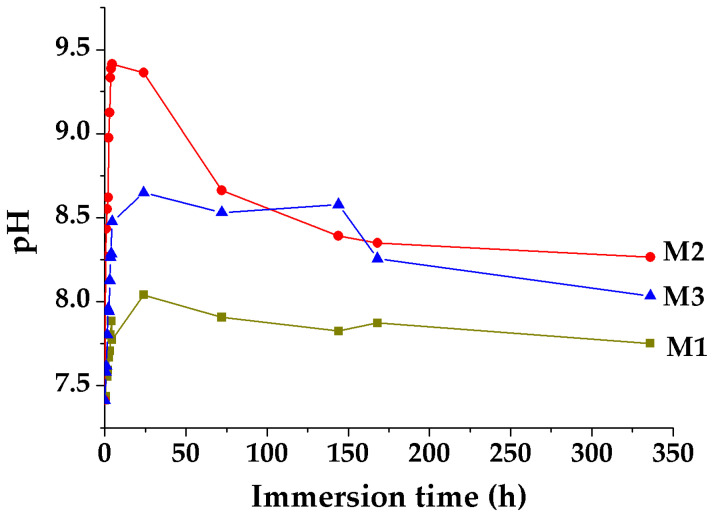
The evolution of the pH solution by immersion of vitreous powders in SBF (pH = 7.4) for 14 days at 37 °C.

By immersing in SBF for 14 days at 37 °C and by monitoring the evolution of pH (Figure 5) it is noticed that the speed of interaction of powders with SBF decreases in the order M2-M3-M1 and this could be explained by the fact that in M2 and M3 masses there is a certain free MgO content (Figure 1b2) that interacts with the aqueous medium; also, for M2, pH value is much higher in the first 24 h after immersion compared to M1 and M3, which could suggest a suitable environment for the precipitation of the apathetic phase. At the same time, there is a higher interaction speed with the SBF of mass M3 compared to mass M1, this being suggested by the higher slope of the pH curve, as well as the maximum reached pH value (after 24 h the pH for M3 is 8.6, and for M1 8.2). At immersion periods longer than 24 h, a decrease of the pH is noticed for all masses below the value indicated by the literature data necessary for the precipitation of hydroxyapatite [24,25], therefore at a small tendency of surface mineralization after 14 days of immersion, especially in the case of masses with Zn^2 +^.

**Figure 6:**
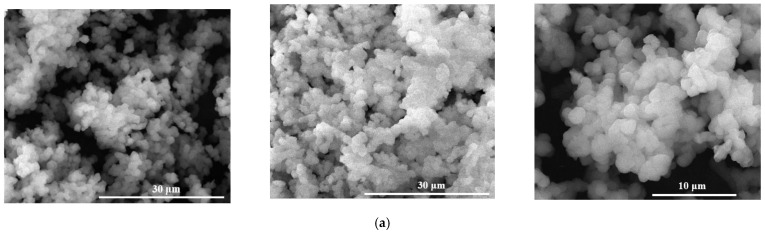

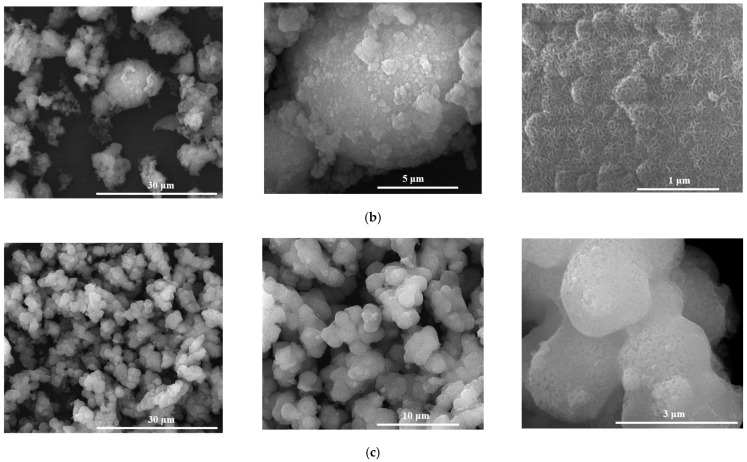
Scanning electron microscopy images for calcined powders immersed for 14 days in SBF (pH = 7.4) at 37 °C ((**a**)—M1, (**b**)—M2, (**c**)—M3).

Scanning electron microscopy images on masses immersed for 14 days in SBF at 37 °C—Figure 6, clearly show an interaction between them by: (i) breaking the existing bridges between particles; (ii) modification of the surface characteristics of the powders; for M2 mass, the presence of platelet crystals is highlighted, agglomerated as spherical aggregates which, according to the literature data, corresponds to the morphology of hydroxyapatite [26,27,28].

**Figure 7:**
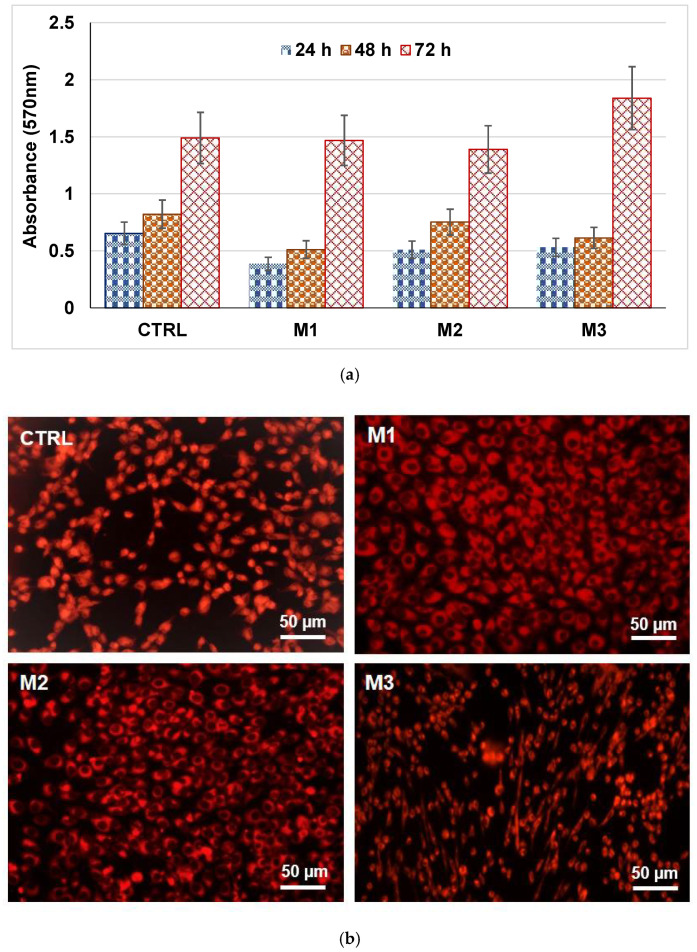
MTT assay (3- (4,5dimetiltiazoliu)-2,5-diphenyltetrazolium bromide) (**a**) for M1, M2 and M3 powders associated with optical fluorescence microscopy images—(**b**) (×20).

Cell biology tests—Figure 7 shows that the analyzed powders are not cytotoxic, regardless of the incubation period. It can also be seen, in the case of powders containing ZnO, that at 24 h and 48 h of incubation, there is a slight decrease in the cell proliferation rate compared to the control sample, more important in the case of M1 powder than M3, and this could be explained by the fact that once in contact with the cells, the powders release a large amount of ions with a stressful effect on them; when incubating with mesenchymal cells for 24 h and 48 h the M2 powder, it can be seen that they develop similarly as in the case of control. At longer incubation times, no significant difference from control is noticed with respect to the growth and development of cells by the contact with these powders, regardless of the mass, because the rate of ion release from the samples decreases and the cells adapt to the new environment proliferating; in addition, for M3 powder, the existence of cytoplasmic extensions is noted (Figure 7b).

**Figure 8:**
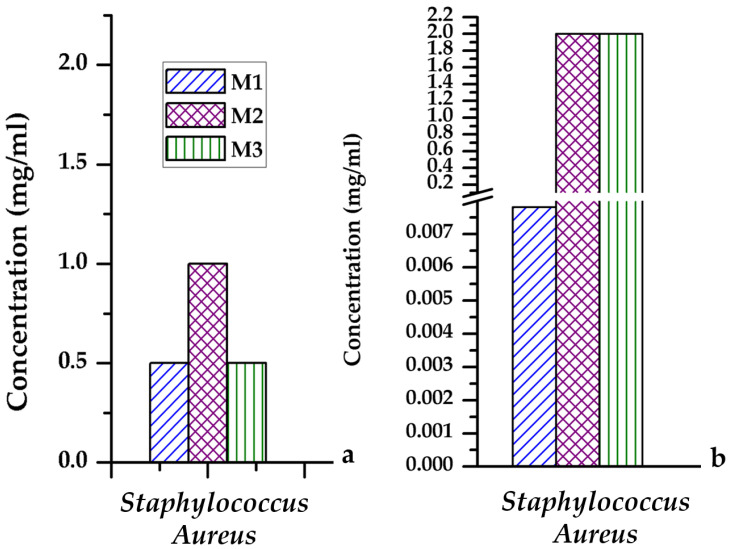
Minimum inhibitory concentration (MIC, (**a**)) and minimum biofilm extinction concentration (MCEB, (**b**)) determined on *Staphylococcus Aureus* for masses M1, M2, and M3.

The antibacterial studies presented in Figure 8a suggest that the efficiency against the development of planktonic cultures for the analyzed powders decreases in the M1-M3-M2 series, so the most effective are the M1 and M3 powders containing Zn^2+^. Regarding the inhibition of biofilm formation—Figure 8b, the M1 sample proves to be the most effective; M2 and M3 powders with Mg^2+^ content show a lower ability to inhibit biofilm formation than M1 powder.

## 3. Conclusions

New vitreous materials from the ZnO(MgO)-CaO-SiO_2_-P_2_O_5_ system were successfully obtained by the sol-gel method by using surfactant—Pluronic F127, starting from the S53P4 system reference (53% SiO_2_, 23% Na_2_O, 20% CaO, 4% P_2_O_5_), in the composition of which Na_2_O was completely replaced with MgO and/or ZnO. Our results show that the interaction speed between the samples and the SBF medium at 14 days after immersion is higher in the case of samples containing magnesium compared to samples containing zinc in composition (M2→M3→M1), which can be explained by the presence of free MgO (results also noticed by X-ray diffraction), that interacts with the aqueous medium. Furthermore, in the case of M2 mass, a modification of the surface characteristics of the powders was observed, the formation of platelet crystals, agglomerated as spherical aggregates which, according to the literature data, correspond to the morphology of hydroxyapatite. The lowest degree of mineralization after 14 days of immersion in SBF at 37 °C was noticed in the case of samples containing zinc.

Biocompatibility tests showed that all three masses are not cytotoxic regardless of the incubation period, and after performing antibacterial studies, the effectiveness of samples against the development of planktonic cultures is highlighted, the highest efficiency being recorded on samples with zinc content.

At long incubation periods, no significant difference from control with respect to cell growth and development is noticed, regardless of mass, in contact with the samples developed in this article, as the rate of ion release from samples decreases, and the cells adapt to the new environment, proliferating.

## 4. Materials and Methods

### 4.1. Materials

For powder synthesis, analytical grade chemicals were used, as SiO_2_, P_2_O_5_, CaO, ZnO and MgO sources, these being purchased from Sigma Aldrich, Steinheim, Germany: tetraethyl orthosilicate (C_8_H_20_O_4_Si, TEOS; 99%), triethyl phosphate (C_2_H_5_O)_3_PO, TEP; >98%), calcium nitrate tetrahydrate (Ca(NO_3_)_2_·4H_2_O; ≥99), zinc acetate dihydrate (Zn(CH_3_COO)_2_·2H_2_O; >98%), and magnesium nitrate hexahydrate (Mg(NO_3_)_2_·6H_2_O; >99), hydrochloric acid (HCl; 1N), isopropyl alcohol (C_3_H_7_-OH; >99%). Additionally, the following chemicals were used: Pluronic^®^ F127 (HO(CH_2_-CH_2_-O)_100_(CH(CH_3_)CH_2_-O)_65_(CH_2_CH_2_O)_100_H; 99%, Sigma-Aldrich, Darmstadt, Germany) and distilled water.

### 4.2. Synthesis of Vitreous Powders

**Figure 9:**
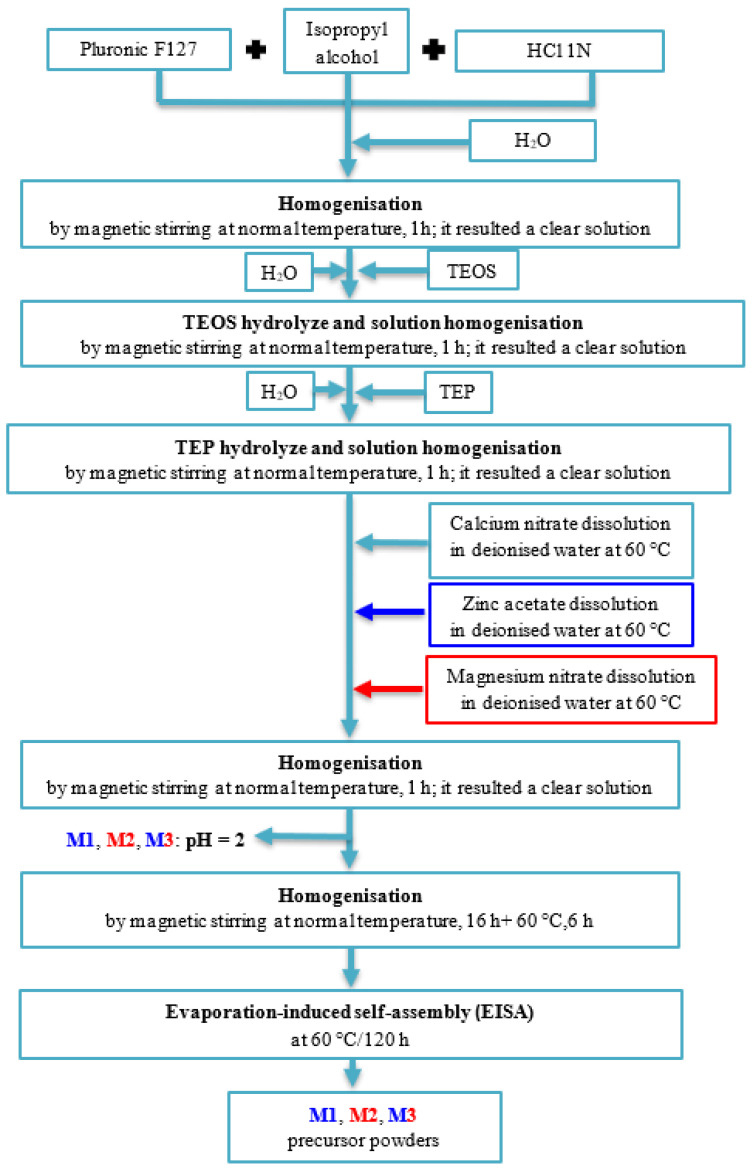
Flow chart of synthesized precursor powders.

The oxidic compositions of synthesized precursor powders by sol-gel method in presence of Pluronic F127 are presented in Table 1. The main steps of this synthesis method are presented in Figure 9; also, the F127:TEOS and F127:H_2_O:C_3_H_7_-OH:HCl weight ratios were 1:4.13 and, respectively, 1:1.33:14.82:0.67, and molar ratios H_2_O:TEOS and H_2_O:TEP were 4:1, and respectively, 3:1.

The dry precursor powders were calcined at 650 °C for 3 h (v = 1 °C/min), in air and then slowly cooled in the furnace in order to obtain the final oxide vitreous powders.

### 4.3. Characterization of Powders

The dry gel-precipitates were characterized by ***complex thermal analyses* *(DTA-TG)*** and ***X-ray diffraction (XRD)*** from phase compositional point of view. Furthermore, the final oxide vitreous powders were characterized for: (i) phase composition by ***X-ray diffraction (XRD)***; (ii) morphological composition by ***scanning electron microscopy (SEM)*** analysis and the ***Brunauer–Emmett–Teller (BET)*** analysis; (iii) dispersion characteristics by grain size distribution by ***laser diffraction analysis***.

Additionally, the powders were evaluated for ***biocompatibility*** properties by in vitro tests and antibacterial properties.

***The thermal analysis (DTA-TG)*** was performed using a Shimadzu DTG-TA-51H equipment (Shimadzu, Kyoto, Japan), in the 20–1000 °C temperature range, with a heating rate of 10 °C/min, in air, using the platinum crucible.

The composition and crystallinity degree of these materials were assessed by ***X-ray diffraction (XRD)***, which was performed using a Shimadzu XRD 6000 diffractometer (Shimadzu, Kyoto, Japan), with Ni-filtered CuK α radiation (α = 1.5406 Å), 2 theta in 5–80° range, with a scan step of 0.02° and a counting time of 0.6 s/step.

The morphology of studied powders was assessed by ***scanning electron microscopy*** ***(SEM)*** using a Quanta Inspect F50 FEG scanning electron microscope, with a resolution of 1.2 nm (Thermo Fisher, Eindhoven, The Netherlands); the powders were covered with a thin gold layer.

The specific surface area and the pores size dimension were assessed by ***the Brunauer–Emmett–Teller (BET) analysis***, which was performed on a Micrometrics Gemini V2 model 2380 (Micromeritics Instruments Corporation, Norcross, GA, USA). The adsorption isotherms were obtained by measuring the amount of gas adsorbed across a wide range of relative pressures at a constant temperature (N2, 77 K and pressure between 780 and 7.8 mmHg). Conversely desorption isotherms were achieved by measuring the gas removed as pressure is reduced.

Particle size distributions of powders were assessed with a Malvern Mastersizer 2000 ***laser diffraction granulometer*** (Malvern Instruments, Malvern, UK).

In vitro **biocompatibility** tests were **MTT assay** and **fluorescent microscopy** for tracing of living cells. Each test was performed in triplicate.

For the **MTT assay**, the human endothelial cells line (EAhy923, ATCC, Manassa, VA, USA) was used to evaluate the biocompatibility of powders. The cells were cultured in DMEM medium (Sigma-Aldrich, St. Louis, MO, USA) supplemented with 10% fetal bovine serum, 1% penicillin, and 1% streptomycin antibiotics (Sigma-Aldrich, St. Louis, MO, USA). To maintain optimal culture conditions, medium was changed twice a week. The biocompatibility was assessed using MTT assay (CellTiter 96^®^ Non-Radioactive Cell Proliferation Assay, Promega, WI, USA). This assay is a colorimetric method that allows quantitative assessment of proliferation, cell viability, and cytotoxicity. The method is based on the reduction of yellow tetrazolium salt MTT (3-(4,5dimetiltiazoliu)-2,5-diphenyltetrazolium bromide) to a dark blue formazan by the mitochondrial enzymes. Briefly, the human endothelial cells were grown in 96-well plates, with a seeding density of 3000 cells/well in the presence of powders for 24–72 h. Then 15 mL Solution I was added and incubated at 37 °C for 4 h. After that, Solution II was added and pipetted vigorously to solubilize formazan crystals. After 1 h, the absorbance was read using spectrophotometer at 570 nm (TECAN, Männedorf, Switzerland).

***The fluorescent microscopy for tracing of living cells*** was used for evaluation of the biocompatibility of powders based on fluorescent microscopy using RED CMTPX fluorophore (Life Technologies, Invitrogen, Carlsbad, CA, USA), a cell tracker for long-term tracing of living cells. The CMTPX was added after 7–28 days of cell culture in the presence of MTA for evaluating the viability and morphology of the endothelial. The CMTPX fluorophore was added in the culture medium at a final concentration of 5 μM, incubated for 30 min in order to allow the dye to penetrate into the cells. Next, the endothelial cells were washed with PBS and visualized by fluorescent microscopy. The photomicrographs were taken with a digital camera driven by Axio-Vision 4.6 software (Carl Zeiss, Jena, Germany). The control cells are represented by the endothelial cells cultivated in the same medium but without the powders.

The ***antibacterial properties*** were determined by using the quantitative minimum inhibitory concentrations (**MIC**-mg/mL, representing the minimum amount of chemical compound capable of inhibiting the growth of microbial cells) assay [29,30,31] and the minimum extinction concentration of the biofilm (**MBC**). The strains used in this study *Staphylococcus aureus* were obtained from the bank of strains of the Microbiology lab, Biology Faculty, University of Bucharest, Bucharest, Romania. The *Staphylococcus aureus* strains were kept in nutrient broth with 20% glycerol at −80°C. For the antimicrobial tests, the microorganisms were inoculated on nutrient agar. The colonies which grew were used to make up suspensions in saline with an optical density of 0.5 McFarland (1 − 3 × 10^8^ UCF/mL).

To work out the MIC and MBC values, a quantitative method based on using binary serial microdilutions in liquid medium (simple broth) was employed. The medium was spread under aseptic conditions in 96-well plates. An aliquot of compound/bioactive nanosystem was added to the first well of each row in the plate so as to obtain a 2 mg/mL concentration in each of these wells. Using a micropipette, 12 serial dilutions were performed across each row starting from the first well (corresponding to a concentration of 2 mg/mL) to the 12th well (whose final concentration was 9.765 × 10^−4^ mg/mL). Once the microdilutions were obtained, 15 μL of microbial suspension of 0.5 McFarland density was added to each well. The plates were then incubated at 37 °C for 24 h, after which the MIC value for each compound/nanosystem was worked out by means of microscopy as the concentration of the well in which bacterial growth had stopped (turbidity of the media levelled off). To distinguish between MIC and MBC, spectrophotometric readings of the liquid bacterial culture were taken at 620 nm, as well as counting the number of viable units (CFU/mL = colony-forming units/mL).

Surface mineralization tests by immersion in SBF (pH = 7.4) were performed at 37 °C for 14 days (ratio ml SBF: mg powder of 11:22 mg), and then washed gently, by immersion in distilled water for 24 h and subsequently dried at 60 °C and analyzed by SEM. At the same time, up to 14 days of immersion of the powders in SBF (pH = 7.4) at 37 °C, the pH of the solution was monitored in order to observe its ability to interact with it.

## Figures and Tables

**Table 1:** Oxidic compositions of studied vitreous powders.

Powders Code	Oxidic Composition (wt%)				
	*SiO*_2_	*CaO*	*P*_2_ *O*_5_	*ZnO*	*MgO*
**M1**	53	20	4	23	-
**M2**	53	20	4	-	23
**M3**	53	20	4	11.5	11.5

**Table 2:** BET surface and average grain dimension of the vitreous powders (calcined powders at 650 °C/3 h).

Powders Code/ Dispersional Characteristic	M1	M2	M3
**S_BET_ (cm^2^/g)**	106.49	164.92	93.87
**d (µm)**	8.37	29.30	11.60
